# Task-Correlated Cortical Asymmetry and Intra- and Inter-Hemispheric Separation

**DOI:** 10.1038/s41598-017-15109-x

**Published:** 2017-11-03

**Authors:** Yaniv Cohen, Donald A. Wilson

**Affiliations:** 1 Department of Child and Adolescent Psychiatry, New York University School of Medicine, New York, USA; 2 Emotional Brain Institute, Nathan Kline Institute for Psychiatric Research, Orangeburg, USA

## Abstract

Cerebral lateralization is expressed at both the structural and functional levels, and can exist as either a stable characteristic or as a dynamic feature during behavior and development. The anatomically relatively simple olfactory system demonstrates lateralization in both human and non-human animals. Here, we explored functional lateralization in both primary olfactory cortex – a region critical for odor memory and perception- and orbitofrontal cortex (OFC) – a region involved in reversal learning- in rats performing an odor learning and reversal task. We find significant asymmetry in both olfactory and orbitofrontal cortical odor-evoked activity, which is expressed in a performance- and task-dependent manner. The emergence of learning-dependent asymmetry during reversal learning was associated with decreased functional connectivity both between the bilateral OFC and between the OFC-olfactory cortex. The results suggest an inter-hemispheric asymmetry and olfactory cortical functional separation that may allow multiple, specialized processing circuits to emerge during a reversal task requiring behavioral flexibility.

## Introduction

Classical examples of lateralized functions are human language and preferential handedness, though there is extensive evidence of asymmetry in cognitive and emotional processes^1–6^. This asymmetry is important for normal function given that deficits in hemispheric asymmetry are associated with impaired cognitive functions such as perception and memory^7–11^. Hemispheric specialization can enhance processing capabilities through specialization and compartmentalization, i.e., creation of new cortical space by reduction of bilateral redundancy^5,12,13^.

In humans, while simple odor detection does not induce asymmetrical cortical activation^14^, lateralization has been demonstrated in more complex olfactory cognitive functions^14–17^. In human subjects asked to recognize or judge the familiarity of odors, fMRI data suggest that these tasks are heavily mediated by right hemisphere structures such as the piriform cortex (PCX, primary olfactory cortex) and orbitofrontal cortex (OFC)^14,18^. Furthermore, damage to the right OFC, but not left OFC impairs odor recognition in humans^19^. In contrast, the ratings of odor hedonics (e.g., pleasant/unpleasant) are more dominated by the left OFC^14,18^.

Despite the potential importance of cortical asymmetry and lateralized disruption in cognitive disorders, there are few rodent models of cortical asymmetry that allow dissection of underlying mechanisms and identification of causal links between asymmetric function (or dysfunction) and cognitive/perceptual olfactory behavior. We recently demonstrated a robust, transient asymmetry in rat anterior piriform cortical (aPCX) function during specific stages of olfactory learning^20^. This asymmetry was expressed both as differential left-right aPCX activation over the course of odor learning, as well as performance- and context-specific changes in bilateral aPCX functional connectivity.

The aPCX receives direct input from the ipsilateral olfactory bulb and has strong reciprocal connections with the ipsilateral OFC^21–23^. Connections via the anterior commissure and corpus callosum allow bilateral communication between these olfactory structures, with for example cells in aPCX capable of responding to odors delivered to either the ipsilateral or contralateral naris^24^. While the aPCX is believed to play a crucial role in odor perception and memory^25,26^, the OFC has been demonstrated to be involved in a number of higher order functions, including those related to reversal learning^27–30^. Reversing the associations of a well learned memory requires significant behavioral flexibility, which we hypothesized might be facilitated by asymmetry in cortical function. Here, we recorded local field potential (LFP) oscillatory activity from bilateral OFC and from aPCX in behaving rats to examine asymmetry in olfactory cortical function, as well as intra- and inter-hemispheric cortical functional connectivity, during initial odor learning and reversal learning. The results suggest that odor learning tasks requiring behavioral flexibility (reversal) are associated with olfactory cortical lateralization.

## Results

**Figure 1:**
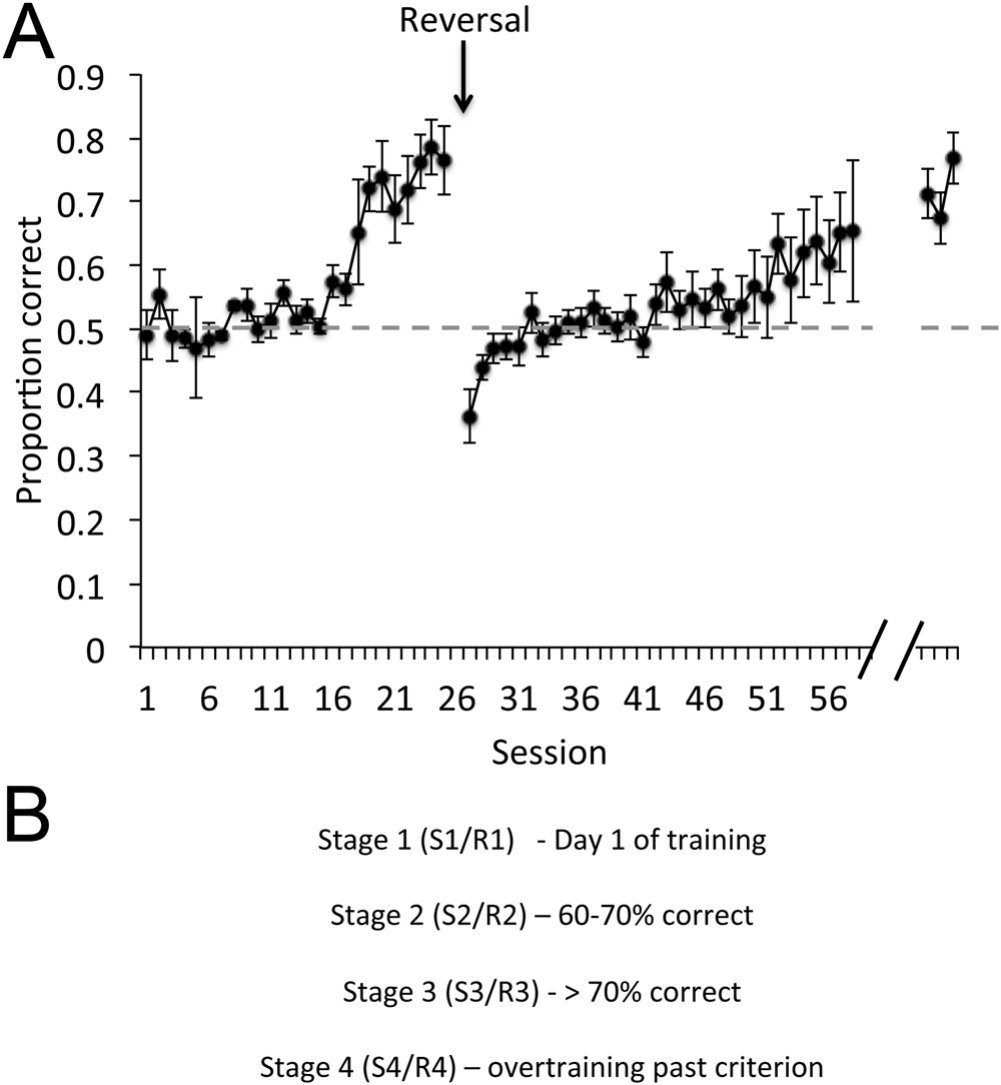
(**A**) Mean (±SEM) initial learning and reversal performance across all rats and sessions. N’s vary across sessions given that rats acquired the task at different rates. (**B**) Operational definitions of conditioning stages during both initial (S1–4) and reversal (R1–4) learning.

Rats were trained in a two-alternative forced choice task for water reward to attain a criterion performance of at least 75% correct on a peppermint vs. vanilla task. They were then implanted with bipolar stainless steel electrodes aimed at both the left and right OFC and a third bipolar electrode aimed at either the left or right aPCX. The electrodes were attached to a connector that allowed telemetry recordings (EMKA) in freely moving animals during each day of subsequent training. Following at least two weeks of recovery, rats were trained with a new odor pair. Odors included peppermint, vanilla, anise, orange (all extracts from McCormick) or overlapping 10 component mixtures of monomolecular components as previously described^31–33^. Rats generated a mean of 221 ± 7 trials/daily session. Rats that successfully reached criterion (75% correct) in the new odor discrimination task then underwent reversal training, where the odor that had originally signaled a reward in the right water port now signaled water reward in the left. LFP data from animals that successfully acquired the initial learning (n = 8) or both tasks (n = 6) to criterion (Fig. 1) were analyzed. Finally, rats were tested for handedness (see Methods). Our results were independent of the handedness expressed by the recorded rats (see Methods).

Given that rats acquired the tasks at different rates (e.g., mean sessions to criterion in initial learning = 14 ± 3, mean sessions to criterion for reversal = 24 ± 4), LFP data were analyzed in a performance-based manner as previously described^20^. For both initial acquisition and reversal, performance stages were defined as 1) initial day of training, 2) 60–70% correct, 3) >70% correct, and 4) overtraining past criterion performance.

### Asymmetry in the PCX

**Figure 2:**
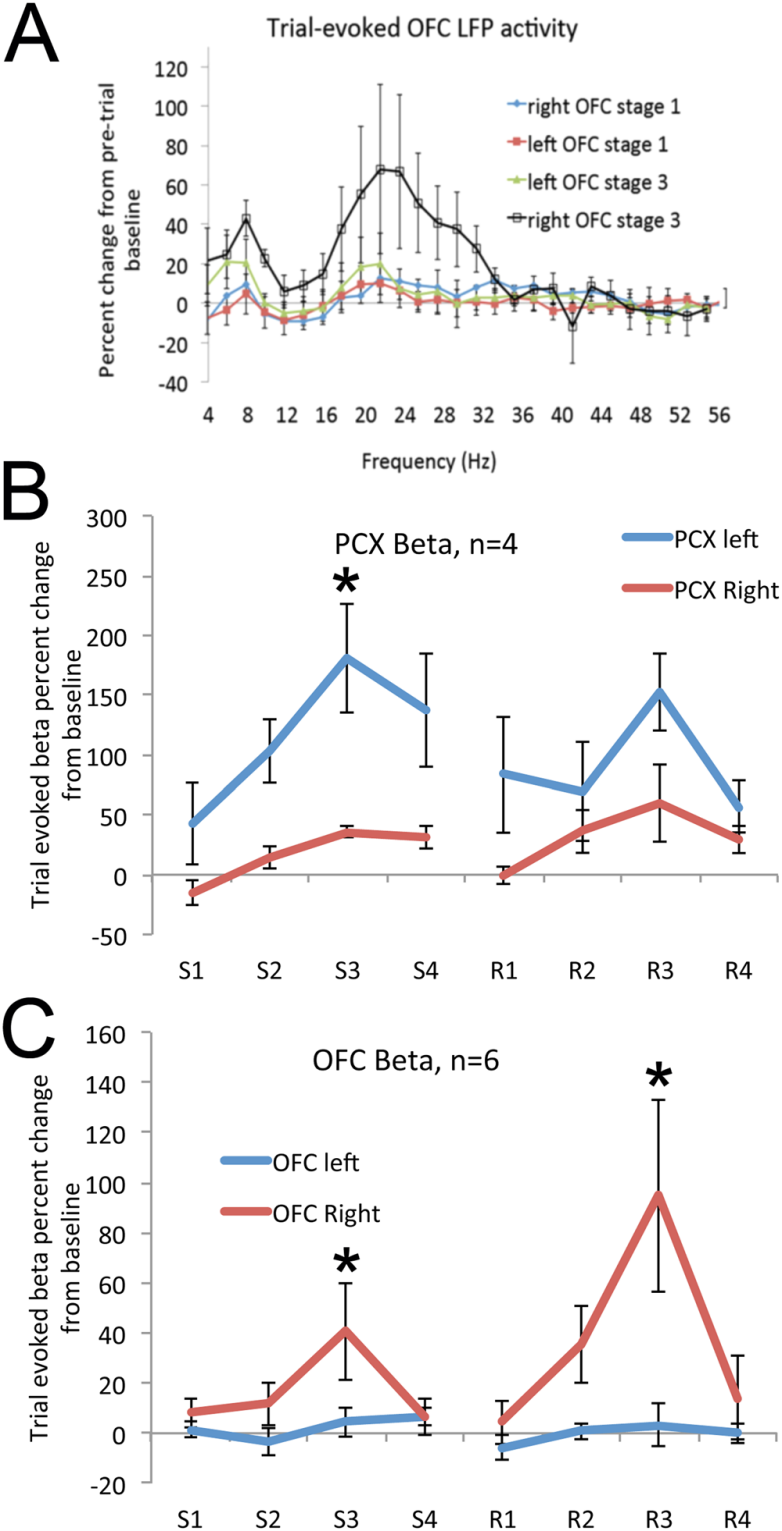
LFP analysis of aPCX and OFC oscillatory activity during the odor sampling period as percent change from pre-sampling activity. (**A**) FFT analysis of bilateral OFC LFP’s on the initial day of training and during stage 3 of initial learning when the animals are reaching criterion. Note the selective rise in beta band power in the right OFC during stage 3. (**B**) Trial-evoked beta oscillations in left and right aPCX over the course of initial (s1-s4) and reversal (r1-r4) training. Performance-defined stages are described in the text. Left aPCX displayed significantly greater beta oscillations during stage 3 of initial learning than the right aPCX. There was no significant asymmetry in the aPCX during reversal learning. (**C**) Trial-evoked beta oscillations in left and right OFC over the course of initial (s1-s4) and reversal (r1-r4) training. The right OFC displayed significantly greater beta frequency oscillations than the left during stage 3 of initial learning and stage 3 of reversal learning. Asterisks signify significant difference between hemispheres, p < 0.05.

Event-related LFP oscillations were analyzed with FFT (3.9 Hz bin width). Analyses were focused on the 1 s period immediately before initiation of a trial by entering the odor sampling port and the 1 s period immediately after entering the odor sampling port^20^. Although a variety of cognitive processes are occurring during these time periods, for simplicity the pre-odor sampling period is referred to here as “spontaneous” activity and the activity during odor sampling as “odor-sampling” related activity. Odor-sampling related activity was expressed as a percent of pre-sampling activity for normalization, and the normalized data was used for statistical comparisons both across hemispheres and across performance stages. LFP oscillation data were binned from the FFT analyses into broader frequency bands, though the major effects observed were within the beta frequency band (15.6–35.11 Hz) (Fig. 2A) which is the focus of the data shown. Beta band oscillations have been widely recognized as a sensitive marker of learning in the olfactory system^34–37^.

As previously reported^20^, during initial learning the left aPCX showed significantly enhanced beta band oscillations during odor sampling compared to the right aPCX (Fig. 2B). This asymmetry was most pronounced during performance stage 3, just as the animals reach criterion (2 × 8 ANOVA, hemisphere × performance stage: main effect of hemisphere, F(1,42) = 10.95, p < 0.05; main effect of stage, F(7,42) = 3.40, p < 0.01). Post-hoc tests (Fisher, p < 0.05) revealed a significant enhancement in the left aPCX beta oscillation power compared to right aPCX during initial learning stage 3. These results replicate our previous observations^20^. In contrast to initial learning, reversal learning resets this asymmetry and lateralized differences in aPCX beta oscillations are much less pronounced during this phase of training. Post-hoc tests revealed no significant difference between the left and right aPCX during any stage of reversal learning.

**Figure 3:**
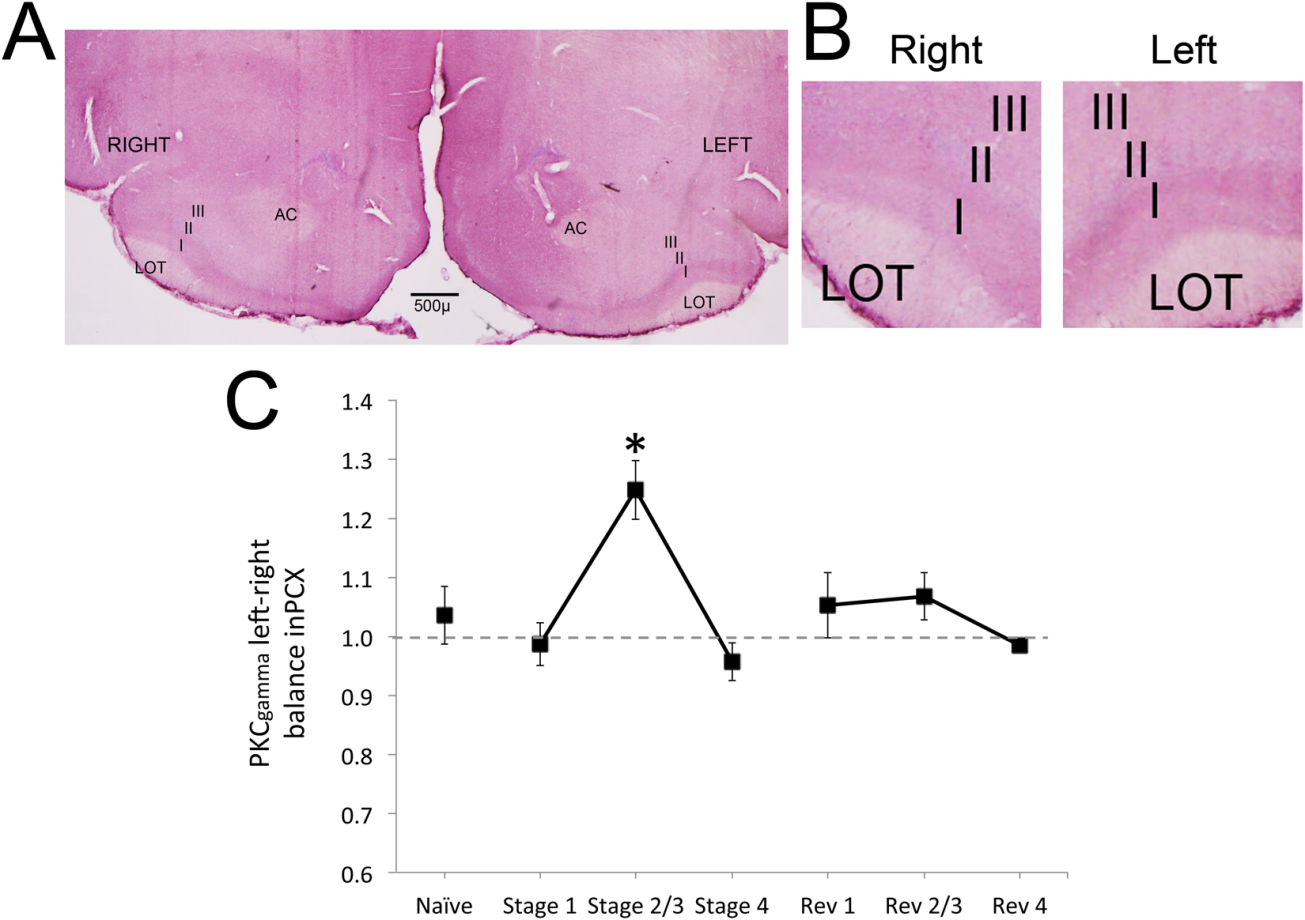
(**A**) Representative PKC_gamma_ immunohistochemistry in bilateral aPCX at Stage 3. (**B**) Side-by-side comparison of Stage 3 aPCX PKC_gamma_ immunohistochemistry. (**C**) Mean (±SEM) asymmetry across initial and reversal learning shows elevated left PKC_gamma_ staining during initial stages 2/3 compared to all other stages. Staining density was quantified in Layer I with optical densitometry (darker staining produced lower values) and compared across hemispheres within each animal (right aPCX/left aPCX, higher ratios signify great staining in the left aPCX). Asterisk signifies significant difference from all other stages, p < 0.05.

If the performance-dependent lateralized changes in aPCX function involve asymmetry in cortical plasticity, we might expect markers of synaptic plasticity to also display performance-dependent asymmetry. Long-term synaptic plasticity is dependent on a variety of receptors and molecular cascades, one of which includes the PKC family. In fact, Olds *et al*.^38^, demonstrated that odor learning enhances PKC translocation to the membrane in the left aPCX more than in the right PCX. Here, we examined PKC_gamma_ levels using immunohistochemistry in the aPCX in animals killed after each stage, or in naïve rats. No LFP recordings were made from these animals. PKC_gamma_ levels were quantified with optical densitometry of immunohistochemical staining in anterior aPCX layer I (minimum of 3 sections/hemisphere/rat), and a ratio of left-right density used to compute a laterality score. As shown in Fig. 3, PKC_gamma_ in the left PCX was significantly enhanced relative to the right, and relative to Naïve animals during stage 2/3 of initial odor learning as the animals approached criterion performance. A 2 × 7 repeated measures ANOVA, hemisphere (repeated) X training stage revealed a significant main effect of hemisphere (F(1,21) = 6.06, p < 0.05), and a significant interaction between hemisphere and performance (F(6,21) = 4.66, p < 0.005). Post-hoc tests revealed PKC_gamma_ labeling was significantly biased toward the left aPCX during Stage 2/3 compared to other stages (Naïve, n = 5, Stage 1, n = 5, Stage 2/3, n = 6, all other stages, n = 3). No such asymmetry was observed in aPCX during reversal learning. These results suggest that the asymmetry in aPCX evoked LFP beta oscillations that emerges during initial learning is associated with asymmetric synaptic plasticity within the aPCX.

### Asymmetry in a higher order olfactory area (OFC)

LFP beta oscillations recorded bilaterally in the OFC also demonstrated asymmetry, though in contrast to the aPCX which showed a left bias, it was the right OFC that demonstrated significantly greater learning associated change than the left (Fig. 2C). A 2 × 8 ANOVA (hemisphere × performance stage) revealed a main effect of hemisphere, F(1,70) = 5.92, p < 0.05; main effect of stage, F(7,70) = 3.83, p < 0.01, and a significant hemisphere x stage interaction, F(7,70) = 3.15, p < 0.01). Post-hoc tests revealed a significant enhancement in the right OFC beta oscillation power compared to left OFC during both initial stage 3 and reversal stage 3. Additional analyses isolated correct from error trials. There was no significant difference in beta oscillations between correct and error trials across stages within hemispheres (within hemisphere analyses, trial outcome X stage, 2 × 8 ANOVA, left OFC, main effect of trial outcome: F(1,48) = 0.04, N.S.; right OFC, F(1,48) = 0.01, N.S.). Furthermore, analysis of just error trials showed a similar emergence of OFC asymmetry with training (error trials only: hemisphere X stage, main effect of side, F (1,48) = 7.38, p < 0.02). This significant OFC lateralization during reversal is in contrast the lack of asymmetry in the aPCX during reversal. No learning dependent change in PKC_gamma_ staining in the OFC was detected (not shown).

Together these results suggest a highly dynamic lateralization of aPCX and OFC cortical function, dependent on performance level and task. We were further interested to know whether this apparent hemispheric specialization was associated with changes in functional connectivity, either between hemispheres or between OFC and aPCX.

### Asymmetry in the functional connectivity either between hemispheres or between OFC and aPCX

**Figure 4:**
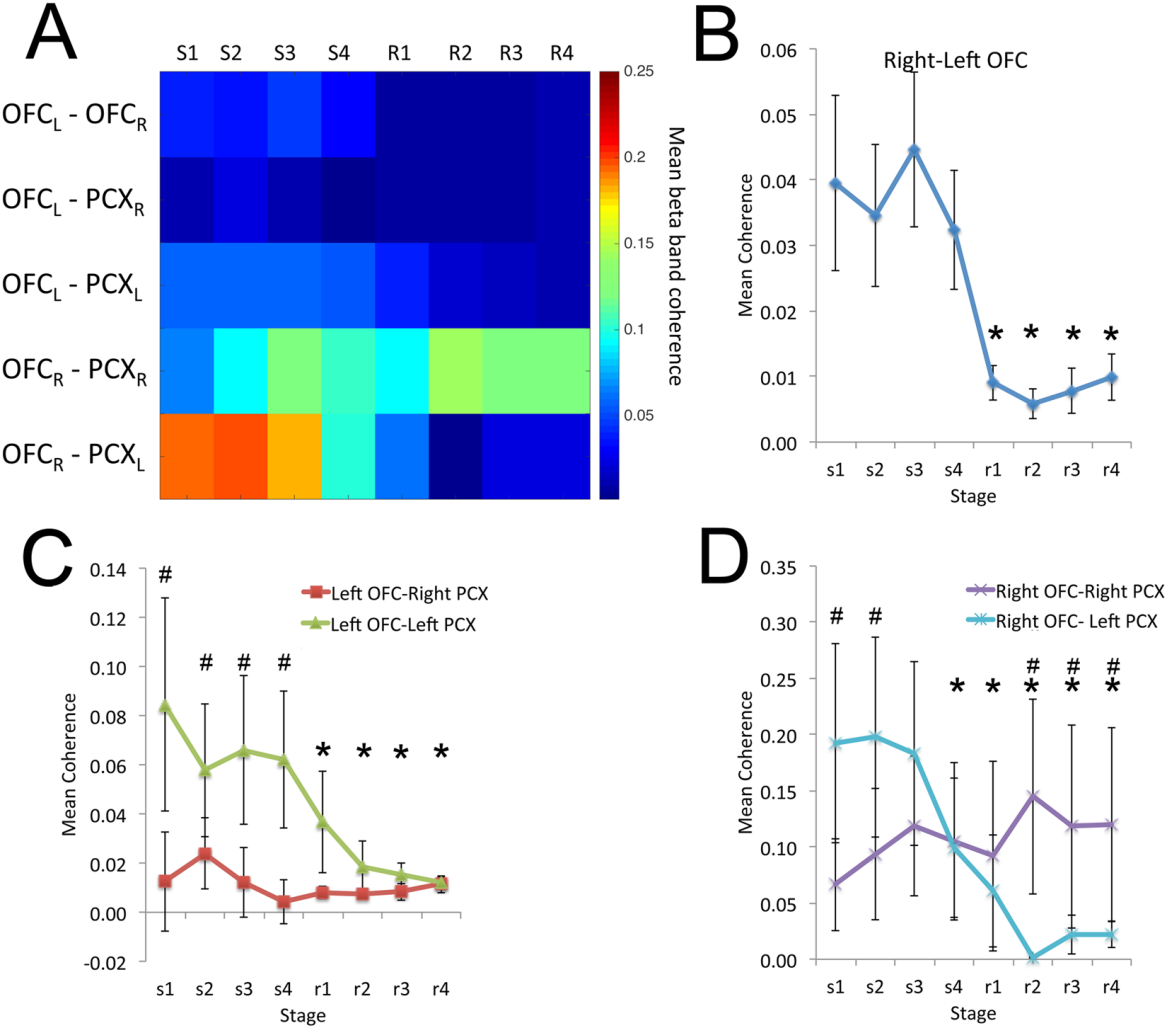
Mean beta band LFP coherence between bilateral OFC and OFC-aPCX as quantified across each training session (initial learning: s1-s4; reversal learning r1-r4). (**A**) Pseudocolor summary of beta band coherence in each tested pathway. (**B**) Bilateral OFC beta band coherence across each performance stage. Reversal training induced a significant decrease in bilateral coherence compared to initial learning in the same animals (asterisk signifies significant decrease from initial Stage 1, p < 0.05). (**C**) Coherence between the left OFC and the ipsilateral and contralateral aPCX. During initial learning there was significantly greater coherence between the left OFC and ipsilateral aPCX than the left OFC and the contralateral aPCX (^#^significant difference between pathways, p < 0.05). This enhanced ipsilateral coherence decreased during reversal conditioning (asterisk signifies significant decrease from initial Stage 1, p < 0.05). No learning associated change was observed in the left OFC to right aPCX pathway at any stage. (**D**) Coherence between the right OFC and the ipsilateral and contralateral aPCX. There was no change in beta coherence between the right OFC and the ipsilateral aPCX at any performance stage. In contrast, the right OFC displayed significantly greater coherence with the contralateral aPCX during initial learning (^#^significant difference between pathways, p < 0.05), which then significantly declined during reversal learning compared to the ipsilateral pathway (^#^p < 0.05) and compared to the first stage of initial learning (*p < 0.05).

LFP coherence in the beta frequency band was quantified over the entire training session(see Methods and^20^). As shown in Fig. 4, OFC bilateral coherence in the beta band significantly decreased during reversal learning compared to initial learning (ANOVA, F(7,49) = 2.21, p < 0.05). Post-hoc tests revealed bilateral OFC coherence during all reversal stages were significantly lower than during initial learning. A similar significant decrease in beta band coherence was observed between the right OFC and contralateral aPCX during reversal learning (2 × 8 ANOVA, hemisphere x stage interaction, ANOVA, F(7,56) = 3.19, p < 0.01). Post-hoc tests revealed right OFC-left aPCX coherence during reversal was significantly lower than during initial learning. No change was observed in coherence between the right OFC and the ipsilateral aPCX. The connectivity between the left OFC and left aPCX was also suppressed during reversal learning, with no change between the left OFC and right aPCX (2 × 8 ANOVA, hemisphere × stage interaction, ANOVA, F(7,56) = 2.63, p < 0.05). Post-hoc tests revealed left OFC-left aPCX coherence during reversal was significantly lower than during initial learning. No stage-dependent change occurred between the left OFC and left aPCX.

**Figure 5:**
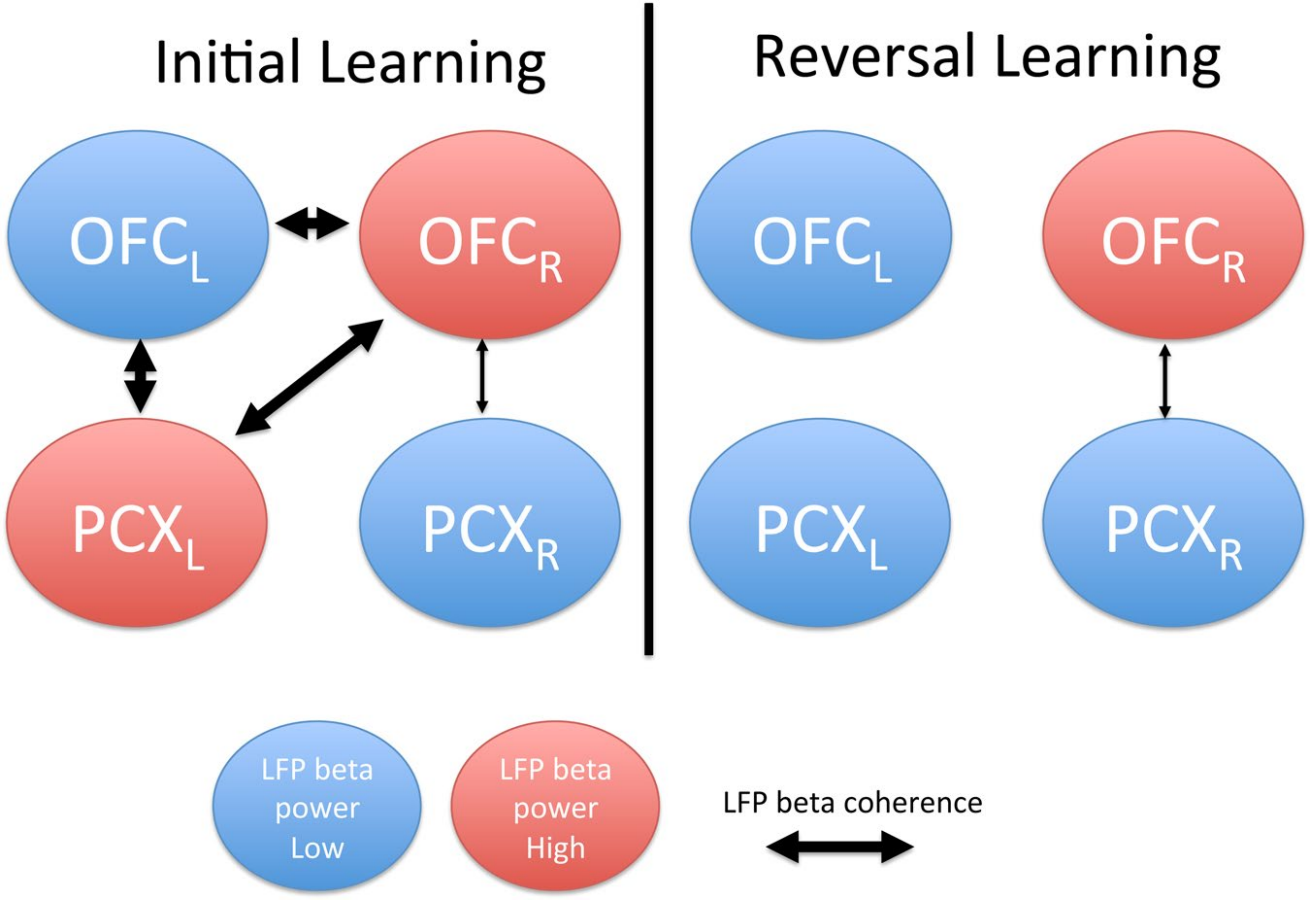
Schematic diagram summarizing learning induced changes in bilateral OFC/aPCX trial-evoked beta oscillations and functional connectivity. The elevation in beta power occurred selectively during stage 3 of either initial or reversal learning. The decrease in functional connectivity was expressed across all stages of reversal learning relative to initial learning.

As summarized in Fig. 5, these results suggest that during initial learning, there is strong network coherence between the bilateral OFC and aPCX cortices, with strongest links between the bilateral OFC and left aPCX. During reversal learning, while OFC-aPCX connectivity is maintained in the right hemisphere, the rest of the network is disrupted, resulting in lateralized, relatively separated, cortical components.

## Discussion

The present data suggest a robust performance and task-dependent asymmetry in the rat OFC and PCX, as well as in the functional connectivity between these cortical structures. These results replicate and extend our previous work on asymmetry in the aPCX to show that, within the same animals, OFC and aPCX display distinct patterns of asymmetry, with a left aPCX bias during initial odor discrimination learning, and a right OFC bias during initial learning and especially reversal learning. The left aPCX bias is associated with enhancement in PKC_gamma_ staining in the left aPCX, suggesting lateralized synaptic plasticity may underlie this form of asymmetry. In addition to the region- and task-specific lateralization, functional connectivity between these structures also varied with task, with significant decreases in bilateral OFC and in OFC-aPCX coherence during the reversal task. Interestingly, our previous work demonstrated a similar reduction in aPCX bilateral coherence during initial learning^20^. By analyzing the data from individual rats, previously^20^ and in this current paper, we argue that intra- and inter- olfactory cortical separation is not simply correlated to the difficult nature the task per se but rather to the flexibility required for task. Reversing of the initial acquired information requires significant behavioral flexibility which expresses in extensive cortical separation. This model reflects non-holistic dynamic computation during flexible olfactory tasks. Overall, functional lateralization can enhance circuit specialization and allow for parallel processing, improving speed and efficiency, especially during tasks requiring behavioral flexibility^5^.

There are a variety of mechanisms that could account for the lateralization and functional isolation of these olfactory circuits, including differential effects of neuromodulators and/or synaptic/cellular plasticity. For example, recent work has demonstrated lateralization in hippocampal contributions to memory in rodents^39,40^, mediated by asymmetrical synaptic plasticity^40^. There may also be evidence of asymmetry in plasticity in the olfactory system. For example, our results here demonstrate an asymmetry in aPCX PKC_gamma_, a marker sensitive to neural plasticity that aligns well with aPCX LFP changes. The PKC changes observed here support an earlier study of odor memory associated PCX lateralization in PKC^38^. Furthermore, two separate studies that investigated learning-induced synaptic strength in the OFC - > aPCX pathway found distinct patterns of learned changes depending on the hemisphere investigated. Following odor-rule learning, there is a potentiation of OFC evoked responses in the ipsilateral aPCX in the right hemisphere^41^, but a depression of OFC evoked responses in the ipsilateral aPCX in the left hemisphere^42^. Importantly, we find no differences in the physiological modifications between left and right handed rats (see Methods). This further supports a behaviorally dependent source for cortical olfactory lateralization in rodents. Further work is required to identify specific mechanisms of dynamic laterality, though these studies suggest asymmetry in neural plasticity may be an important factor in odor processing and memory.

Together, the results suggest that the rodent olfactory cortex displays task- and performance-dependent asymmetry, similar to that observed in the human olfactory system. The lateralization of aPCX corresponds to changes in expression of the plasticity associated PKC_gamma_. OFC lateralization appears most pronounced during reversal learning, a task known to be OFC-dependent^29^. As this area-specific lateralization emerges during reversal learning, there is a concomitant functional de-coupling of these regions, allowing hemispheric and regional specialization. These findings open the opportunity for future studies of olfactory cortical asymmetry contributions to odor memory in condensed non-spatial paradigms, for example as in odor-fear conditioning.

## Methods

### Subjects

Male Long-Evans hooded rats (250–450 g) were used as subjects. Animals were housed individually on a 12 hr light/dark cycle, with food and water available *ad libitum* unless specifically noted. Tests were performed during the light portion of the day/night cycle. All handling, housing and experimental procedures were approved by, and performed in accordance with the Nathan Kline Institutional Animal Care and Use Committee guidelines (IACUC protocol number AP2014–489).

### Paw preference test

Previous research indicates that there is an equal distribution of left or right handedness in rodents^43,44^ while Tang and Verstynen^45^ mention earlier studies suggesting a slight right-handedness preference. Here, handedness was tested using a standard reaching task. 10cc syringe with tip cut off to 4 ½ cm, syringe plunger, Froot Loops cereal pieces (about 0.05 to.08 grams each). Testing occurred in the rats’ homecage. With each rat, the first few cereal bits were pushed to the end of the syringe to allow the rat to get the treat easily with their mouth. Gradually the cereal was kept further back to 3 cm so that the rat would have to use his paw to grab it, allowing us to determine their handedness. A rat was classified as right or left handed if it reached at least 75% with one hand, a criterion which all animals attained (see Supplemental Fig. 1).

### Chronic electrophysiology

Bipolar stainless steel electrodes (127 μm diameter wire, minimal distance between the two tips of approximately 20 μ) were implanted bilaterally in the Ventralis lateralis pars oralis (VLO; coordinates from Bregma - Anterior: 3.2 mm, Lateral: ± 2 mm, Ventral: 4.2 mm) and in layer III of the left or right anterior PCX (aPCX; coordinates from Bregma - Anterior: 2 mm, Lateral: ±4 mm, Ventral: 6 mm) under isoflurane anesthesia. Electrodes and a ground lead over posterior cerebral cortex were cemented to the skull with dental acrylic and attached to a connector. Following at least 2 weeks of recovery, the head connector was attached to a telemetry transmitter (EMKA Technologies) which allowed free movement and performance in the odor discrimination task while recording LFP’s. The bipolar differential recording signals were amplified (4000x) and transmitted at 1 KHz to the telemetry receiver, and then fed to an analog to digital converter and acquired and analyzed with Spike2 software (Cambridge Electronic Design). LFP recordings were synchronized with simultaneously recorded behavioral markers (sampling and water port entries and exits) in the operant chamber (Vulintus). Following the termination of testing, animals were overdosed with anesthetic, transcardially perfused with 10% formalin, and brains sectioned to confirm electrode placements.

### Behavioral training and testing

Animals were given limited access to water during behavioral training and body weight monitored to ensure no animal lost more than 15% of initial body weight. All animals gained weight over the course of training. Odor discrimination was assessed in a two-alternative choice, Go-Left, Go-Right odor discrimination task for water reward. Animals received 30 min training sessions, 5 days/week. Individual animals received a single training session/day and generated >100 trials/session. Trials were initiated by a nose poke in the odor port (at least 350 ms, with odor onset beginning 100 ms after poke onset). Odor delivery terminated on nose withdrawal from the port. Water reward was delivered, depending on odor identity, upon a correct choice of the left or right reward port. All the animals were first trained on a vanilla versus peppermint discrimination until criterion (performance >75%) was attained. Animals reached this criterion in (mean ± SD) 14 ± 3 d.

Following criterion performance in the vanilla–peppermint discrimination, animals were anesthetized and bilateral electrodes were implanted as described above. The animals were allowed at least 2 weeks to recover, and then began training with a new odor pair. Odors included anise vs. orange or an odor mixture discrimination. The 10 component odorant mixtures which have been described in detail elsewhere^31–34^, and included: isoamyl acetate (100PPM), nonane (100PPM), ethyl valerate (100PPM), 5-methyl-2-hexanone (100PPM), isopropylbenzene (100PPM), 1-pentanol (100PPM), 1,7-octadiene (400PPM), 2-heptanone (100PPM), heptanal (100PPM), 4-methyl-3-penten-2-one (100PPM). For reversal mixture discrimination training, we used the same pair of odors but in a reversed direction from the initial learning. Performance on the task was divided into stages based on individual ability. For both initial acquisition and reversal, performance stages were defined as 1) initial day of training, 2) 60–70% correct, 3) >70% correct, and 4) overtraining past criterion performance.

### LFP data analyses

Bilateral OFC and aPCX LFP data were analyzed for each animal from a single training session within each stage of behavioral performance as previously described^20^. Briefly, analyses included the 1s period immediately before entering the odor sampling port and the 1s period immediately after entering the odor sampling port regardless of the outcome of the trial – correct, error or aborted trial. Although a variety of cognitive processes are occurring during these time periods, for simplicity the pre-odor sampling period is referred to here as “spontaneous” and the activity during odor sampling as “odor-sampling” related activity. The 1 second sampling periods were chosen to allow sufficient data of even low frequency oscillations for accurate Fast Fourier Transform (FFT) analyses (3.9 Hz resolution). Odor-sampling evoked activity was expressed as a percent of spontaneous activity for normalization, and the normalized data was used for statistical comparisons both across hemispheres and across learning stages. LFP oscillation data were binned from the FFT analyses into broader frequency bands, including theta (3.9–11.7 Hz), beta (15.6–35.11 Hz), low gamma (39.06–50.78 Hz) and high gamma (70.31–101.56 Hz) bands.

LFP coherence between the left and right OFC and between the OFC and ipsilateral and contralateral aPCX was also assessed over the entire sessions (30 min). The coherence was determined using a “cohere” script function within Spike2 at 5-Hz resolution.

### PKC_gamma_ Immunohistochemistry and quantification

A group of rats separate from those used for electrophysiology were trained as described above and killed after attaining a specific performance stages, or were killed as naïve rats. Rats were transcardially perfused with 4% paraformaldehyde and post-fixed in glycerol-formaldehyde before sectioning at 40 µm. Standard immunohistochemistry was performed for PKC_gamma_ (primary antibody AbCam, ab71558) and visualized with Vector VIP peroxidase substrate. Images were taken of bilateral aPCX and optical density of staining in Layer I was determined using ImageJ image analysis software. Low values represented higher levels of staining. The right/left ratio was determined as a metric of PKC_gamma_ asymmetry in PCX at each performance stage.

## Electronic supplementary material

Supplementary Figure 1
