# Publisher Correction: Task-Correlated Cortical Asymmetry and Intra- and Inter-Hemispheric Separation

**DOI:** 10.1038/s41598-018-20357-6

**Published:** 2018-02-02

**Authors:** Yaniv Cohen, Donald A. Wilson

**Affiliations:** 1 Department of Child and Adolescent Psychiatry, New York University School of Medicine, New York, USA; 2 Emotional Brain Institute, Nathan Kline Institute for Psychiatric Research, Orangeburg, USA

Correction to: *Scientific Reports* 10.1038/s41598-017-15109-x, published online 03 November 2017

The original HTML version of this Article contained a typographical error in the publication date ‘03 November 2017’ which was incorrectly given as ‘06 November 2017’. This has now been corrected in the HTML version of the Article.

