# Supplementary material for: Task-Correlated Cortical Asymmetry and Intra- and Inter-Hemispheric Separation
Source: Sci Rep. 2017 Nov 3;7:14602. doi: 10.1038/s41598-017-15109-x (PMC5668373; doi:10.1038/s41598-017-15109-x)

Supplementary Information

TASK-CORRELATED CORTICAL ASYMMETRY and INTRA- and INTER-HEMISPHERIC SEPARATION

Yaniv Cohen and Donald A. Wilson

Supplemental Figure 1

Summary of handedness in all animals trained/tested here. All animals expressed at least a 75% bias toward one hand. See Methods for details of behavioral assay.

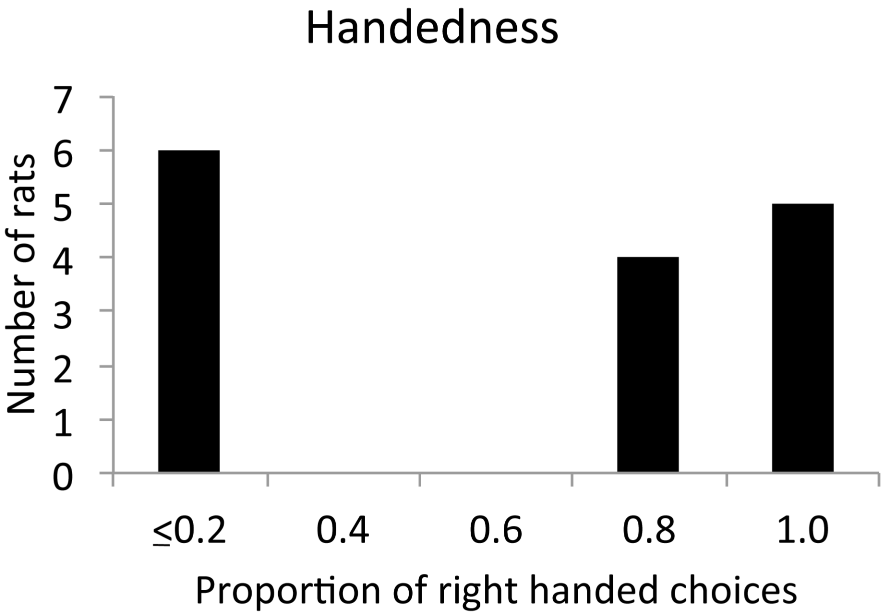

Supplement: Supplementary file 1 — Supplementary Figure 1 [file 41598_2017_15109_MOESM1_ESM.pdf]
